# Tritium in vegetation at various types of radioactive contamination sites under arid climate conditions

**DOI:** 10.1371/journal.pone.0339645

**Published:** 2026-01-27

**Authors:** Natalya Larionova, Lyubov Timonova, Anna Toporova, Pavel Krivitskiy, Vasiliy Polevik, Almira Aidarkhanova, Ainur Mamyrbayeva, Аssan Aidarkhanov

**Affiliations:** 1 Institute of Radiation Safety and Ecology, National Nuclear Center of the Republic of Kazakhstan, Kurchatov city, Kazakhstan; 2 National Nuclear Center of the Republic of Kazakhstan, Kurchatov city, Kazakhstan; 3 Shakarim State University of Semey, Semey city, Kazakhstan; University of South Carolina, UNITED STATES OF AMERICA

## Abstract

This article presents research findings on tritium (^3^H) in plants at the Semipalatinsk Test Site (STS), including the perimeter of the ‘Degelen’ site, the riverside zone of the Shagan River, and a conditionally ‘background’ area—the southeastern part of the STS (SEP). The ^3^H content in plants was determined in tritiated free water (TFWT) and organically bound ^3^H (OBT). Plant samples were measured using an ultra-low-level liquid scintillation spectrometer. Maximum ^3^H concentrations in plants were detected at the ‘Degelen’ site (TFWT up to 51 ± 5 kBq/kg, ОBТ up to 15 ± 1 kBq/kg) and Shagan River (TFWT up to 39 ± 4 kBq/kg, ОBТ up to 13 ± 1 kBq/kg). The presence of ^3^H was recorded not only at underground nuclear sites but also in the conditionally ‘background’ area (TFWT up to 0.18 ± 0.02 kBq/kg, ОBТ up to 0.088 ± 0.028 kBq/kg). Research suggests that water plays a key role in ^3^H migration processes in the natural system of interest. The OBT/TFWT values for the study area varied on average from 0.3 to 0.7. The dose rate of internal irradiation of the studied plants varied from 6.3 × 10^−6^ (SEP) to 1.2 × 10^−2^ µGy/day (at the boundary of the ‘Degelen’ site), which is significantly lower than the safe exposure level for biota.

## 1. Introduction

Tritium (^3^H), a radioactive isotope of hydrogen with a mass number of 3, is a pure β-emitter with a half-life of 12.32 years. Tritium is formed in the upper atmosphere as a result of the interaction of cosmic radiation with the nuclei of nitrogen, oxygen, argon, and other atoms. It is also produced in the lithosphere and hydrosphere through neutron interactions with lithium in the Earth’s crust. Currently, ^3^H is present in the environment from both natural and artificial sources. An estimated annual production rate of tritium is 1.48 × 10^17^ Bq1 and the global inventory of tritium from natural sources is estimated to be about 1–3 x 10^18^ Bq [1–3].

**Fig 1:**
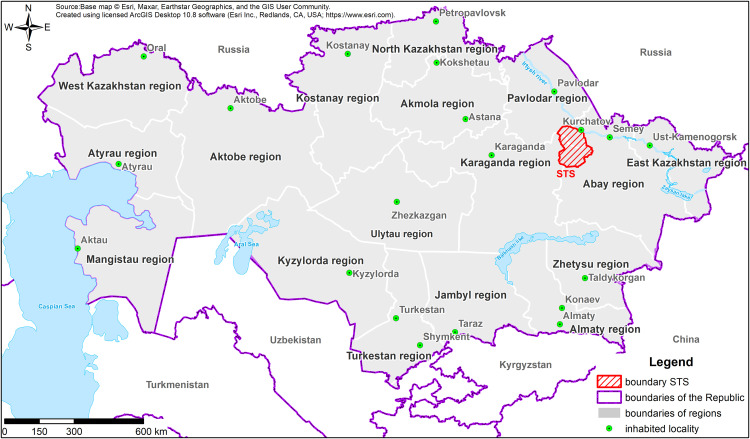
Location of the Semipalatinsk Test Site [15].

The nuclear industry, namely nuclear power plants (NPPs), accounts for a high level of emissions and discharges of man-made ^3^H into the environment [4,5]. Reprocessing plants can emit approximately the same amount or more than entire reactor fleets, but this depends on the capacity and volume of fuel processed. Emissions are released into liquid streams after cleaning and dilution. Heavy-water CANDU reactors have the highest regular ^3^H emissions of all reactor types. However, these volumes are within regulated limits and are monitored by regulators. Light-water reactors produce the least noticeable ^3^H emissions, tens to hundreds of times less than CANDU reactors [6–8]. A distinct category comprises experimental studies involving ^3^H reactors, in which ^3^H is deliberately produced to investigate material selection for protective coatings in thermonuclear reactors [9–13]. However, the greatest release of ³H into the environment occurred with the onset of nuclear and thermonuclear tests [14]. A significant proportion of ^3^H was produced during nuclear tests at the former Semipalatinsk Test Site (STS), located in the Republic of Kazakhstan (Fig 1).

To date, activity at the STS involves the study of a wide range of radionuclides in various environmental media [15–18]. ³H, in turn, has been studied as an indicator of nuclear tests [19] and for assessing groundwater contamination based on its content in vegetation [20]. Separate studies have focused on ³H content in air, soil, and vegetation cover [21–23], as well as the impact of ³H on the morpho-anatomical structure of plants [24]. Despite numerous studies, the STS area remains of particular interest with respect to ³H, as concentrations in certain locations exceed radioactive waste levels. For example, the concentration level of ^3^H in the water of the Shagan River was recorded at up to 400 kBq/kg and in the adit waters of the ‘Degelen’ site – up to 200 kBq/kg [25–27].

Despite the global decline in ^3^H levels following the implementation of nuclear test restrictions, its persistence and variability continue to pose a significant environmental concern [28]. The migration of ³H within environmental components involves complex, long-term, and multi-stage processes that depend on its spatial distribution, seasonal and interannual variability, weather conditions, and nonuniform distribution across different types of ecosystems. The distribution of ³H in the ‘water–soil–air’ system has been examined at various plots within the STS, which are characterised by differing natural conditions and different types of nuclear tests [29]. For example, differences in natural conditions: relief differences (mountains, plains), differences in water resources (groundwater level), soil, geological and biological (vegetation) differences.

Plants are no less important environmental components in terms of ^3^H content [30,31]. When assessing ^3^H in plants, it is necessary to account for its various chemical forms [32], particularly tritiated free water (TFWT) and organically bound ^3^H (OBT). This study continues previous research conducted at various plots within the STS, presenting TFWT and OBT data aimed at comparing plant ^3^H levels in abiotic environmental components, including water, soil, and air [29].

## 2. Materials and techniques

### 2.1 Field work

**Fig 2:**
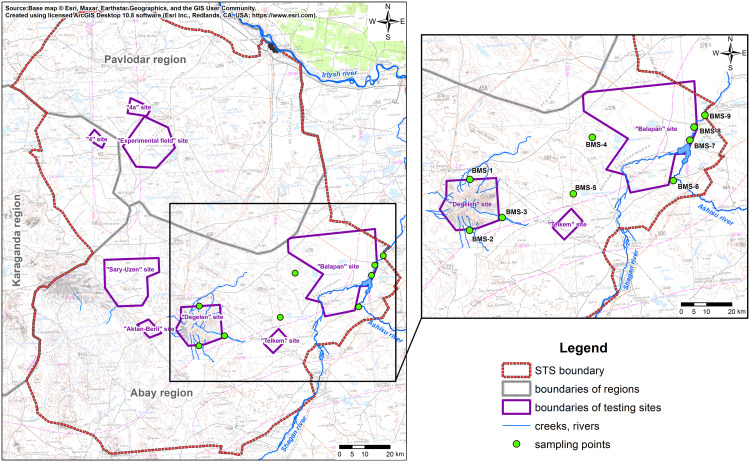
General schematic arrangement of the BMS sites [29].

Research sites were selected based on differences in soil and botanical–geographical zoning, as well as on variations in the nature of ^3^H contamination in environmental components. A total of nine sites, designated biological monitoring sites (BMSs), were selected for the study. The locations are shown in Fig 2.

Research sites were selected along the border of the ‘Degelen’ site and in the riverside zone of the Shagan River with a high ^3^H content in environmental compartments of interest. A conditionally ‘background’ area in the southeastern part of STS (SEP) was also included, where ^3^H levels in environmental compartments remain within background ranges.

At the ‘Degelen’ site, research was conducted on radioactive water streams that had flowed beyond its boundaries (Fig 2). Research areas were located in the bed of three water streams: the Karabulak Creek, which is hydrologically linked to water streams flowing from adits 504 and 511 (BMS-1); the Baytles Creek, originating from the adit 176 water stream (BMS-2); and the Uzynbulak Creek, which originates from the adit 177 water stream (BMS-3).

Research sites at the ‘Degelen’ site are characterised by thin meadow soils (no more than 40–45 cm in streambeds and 20–25 cm in the riverside zone), with a sufficient amount of humus (up to 20%), and well-leached of readily soluble salts and carbonates. The bulk of plant biomass is concentrated in floodplains of watercourses, where diverse meadow vegetation thrives due to additional moistening. Dominant species include fireweed (*Chamaenerium angustifolium*), thistle (*Cirsium arvense*), tansy (*Tanacetum vulgare*), reed grass (*Calamagrostis arundinacea*), nettle (*Urtica dioica*), veronica (*Veronica spuria*), mint (*Mentha interrupta*), sorrel (*Rumex confertus*), geranium (*Geranium collinum*), burnet (*Sanguisorba officinalis*), and larkspur (*Delphinium dictyocarpum*), among others. Shrub thickets consisting of willow (*Salix triandra, S. viminalis*) and brier (*Rosa spinosissima*) grow along the watercourses, while cane thickets (*Phragmites australis*) are widespread in areas with the highest moisture levels (Fig 1 in S1 Appendix).

^3^H distribution in environmental compartments of the riverside zone along the Shagan River was assessed at three sites located downstream from Atomic Lake. The selected sites were characterised by maximum ^3^H concentrations in the water due to the discharge of contaminated groundwater from the underground nuclear test sites at the ‘Balapan’ site [33]. i.e., the BMS-7 site (2 km from Atomic Lake on the left bank), the BMS-8 site (5 km from Atomic Lake on the left bank), and the BMS-9 site (near the bed outlet beyond the ‘Balapan’ site on the right bank). The location of study sites in the riverside zone of the Shagan River is shown in Fig 2.

Light chestnut soils are characteristic of the Shagan impact zone. The plant cover of the bed comprises aquatic and aquatic-riverside species, including cane (*Phragmites australis*), glasswort (*Salicornia europaea*), plantain (*Plantago tenuiflora*), and wild rye (*Leymus angustus*). The terrace comprises steppe cenoses: mat grass (*Stipa sareptana*), sheep’s fescue (*Festuca valesiaca*), liquorice (*Glycyrrhiza glabra*), wheat-grass (*Agropyron cristatum*), wormwood (*Artemisia terrae-albae, A. gracilescens*), cheegrass (*Achnatherum splendens*), wild rye (*L. angustus*), and meadowsweet (*Spiraea hypericifolia*). The transitional scarp between the bed and the terrace, exhibiting plant succession, is often covered in ruderal species: bindweed (Convolvulus arvensis), peppergrass (*Lepidium latifolium*), and hyssop (*Hyssopus macranthus*) (Fig 2 in S1 Appendix).

Research sites in the SEP were near the Balapan site (BMS-4, BMS-5, and BMS-6), where water bodies and a large amount of coastal vegetation were found. According to the results of comprehensive radiological studies, this area has been determined as not susceptible to radioactive contamination [34]. A schematic map of the research site locations is presented in Fig 2.

**Fig 3:**
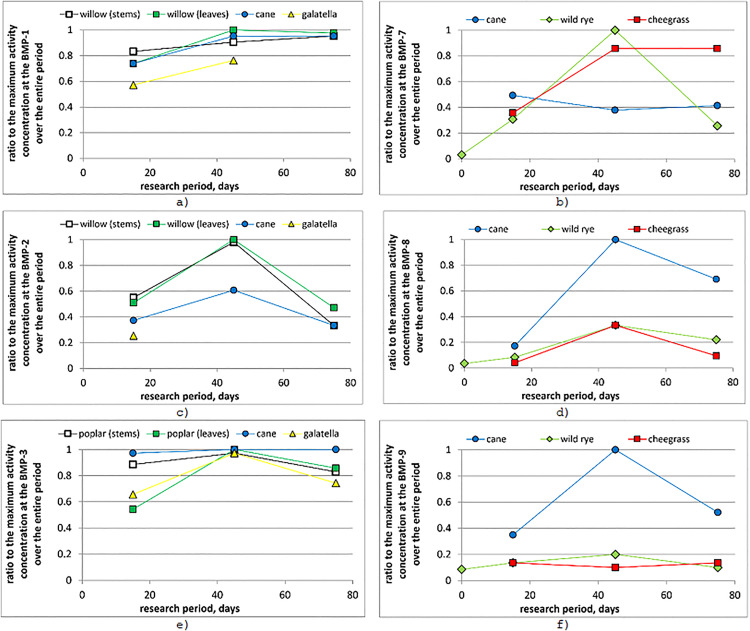
Relative tritiated free water (TFWT) concentrations at the boundary of the ‘Degelen’ site: BMS-1 (а), BMS-2 (c) and BMS-3 (e) and along the coastal area of the Shagan River: BMS-7 (b), BMS-8 (d), and BMS-9 (f).

The plant cover of SEP is classified as dry steppe vegetation on light chestnut soils and is characterised by the presence of saline soils. The vegetation includes a complex of gramineous-sandy needle grass-absinthial associations: mat grass, *(Stipa sareptana)*, sheep’s fescue *(Festuca valesiaca)*, june grass *(Koeleria cristata),* and wormwood *(Artemisia terrae-albae, A. gracilescens)*, among others. Vegetation on saline soils includes glasswort *(Salicornia europaea),* plantain *(Plantago tenuiflora)*, biyurgun *(Anabasis salsa),* and sea lavender *(Goniolimon speciosum),* among others. Cheegrass *(Achnatherum splendens)* and wild rye *(Leymus angustus)* are also present (Fig 3 in S1 Appendix).

Plant sampling was conducted in summer (June, July, August). Additionally, several samples were collected in May (in the coastal area of the Shagan River and SEP). The phreatophytes, willow and poplar (separate stems and leaves) and cane, as well as a meadow species, galatella, were selected as the study plant species at the border of the ‘Degelen’ site. In the coastal area of the Shagan River, cane growing in water, as well as wild rye and cheegrass growing on land, were collected. In SEP, mat grass and cheegrass were collected; due to the lack of cheegrass at the BMS-5 site, meadowsweet was collected instead. Samples of herbaceous plants consisted of the aboveground part of shrubs that had grown in the current year. The area of the research plot for plant sampling was 2–4 m^2^, and the weight of the samples was 300–500 g. The plants were collected in clear (not rainy) weather. All vegetation samples were sealed in double plastic bags (to minimise sample contact with the surrounding air) and then frozen.

### 2.2 Laboratory work

**Fig 4:**
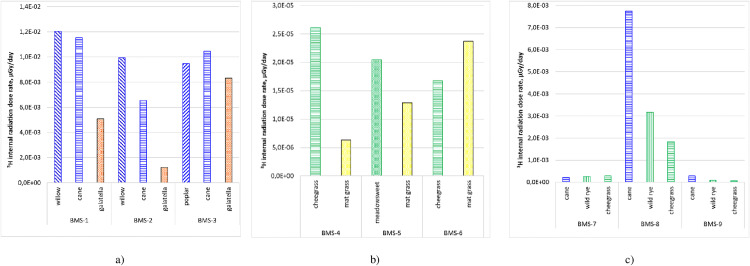
Internal radiation dose rate of terrestrial plants at the boundary of the ‘Degelen’ site (а), southeastern part of the Semipalatinsk Test Site (SEP) (b), and along the coastal area of the Shagan River (c).

Plant samples were prepared to determine the activity concentration of ^3^H in the form of TFWT and OBT, in accordance with Innovative Patent no 29721 [35]. TFWT was extracted using a specialized unit consisting of a sealed transparent chamber for placing the plant sample, connected to a cooled metal surface and a receiver for condensate extracted from the sample. To measure the activity concentration of ^3^H in OBT, dry plant samples were combusted in a Sample Oxidizer A307 unit (Revvity, Waltham, MA, USA). According to the operating principle of the setup, burning the sample leads to the oxidation of all hydrogen isotopes (including tritium) and the formation of superheavy (tritium) water. Due to the high combustion temperature, this water is in a gaseous state. Condensing in the air condenser, tritium steam settles in drops on its walls, and the resulting tritium water flows into the corresponding counting (measuring) container. The mass of the sample to be burned was 1–2 g. The resulting water was then processed for β-spectrometric analysis of ^3^H activity concentration. The plant sample preparation units are shown in Fig 4 in S1 Appendix.

The determination of ^3^H activity concentration in all prepared samples was conducted as described in ISO 9698:2019 [36]. Briefly, a 5-mL aliquot was taken from each sample, and Ultima Gold scintillator (Revvity) was then added in a 1:3 mL ratio. Thereafter, each sample was shaken until a homogeneous mixture was obtained (Fig 5 in S1 Appendix). The measurements were performed using the liquid scintillation technique on a ‘TRI-CARB 2900 TR’ β-radiometer (Revvity) (Fig 6 in S1 Appendix).

The activity concentration of ^3^Н in plants (НТО and OBT) was calculated using the following formula:


$$A_{plant.}=\;\frac{CPM\;-\;{CPM}_{fon}\;}{V_{sample}\;\cdot \;\text{6}\text{0}\;\cdot \;Eff}\;\cdot \text{1},\text{0}\text{0}\text{0},\;Bq/kg,$$ (1)

where CPM is the number of pulses per minute recorded by the spectrometric equipment for the measured sample, cpm; CPM_fon_ is the number of pulses per minute recorded by the spectrometric equipment for the background sample (scintillator and distilled water), cpm; 1,000 is the conversion factor from mL (mg) to L (kg); V_sample_ is the volume (subsample) of the measured sample (5 mL); 60 is the conversion factor for minutes to seconds (from cpm to cps), since 1 Bq corresponds to one radioactive decay per second; Eff is the ^3^H registration efficiency, determined by the formula:


$$Eff=\frac{CPM-\;{CPM}_{fon}}{A_{known}\;\cdot \text{ 60}}$$ (2)

where A_known_ is the known activity of the calibration ^3^Н source, Bq. The minimum detectable activity (MDA) for the determination of ^3^H in vegetation samples was calculated using the formula:


$$MDA=\text{2}\cdot \;\frac{\sqrt{}({CPM}_{fon}\;\cdot \;t)}{t\;\cdot \text{ 60 }\cdot \;Eff\;\cdot \;V_{sample}}\;\cdot \text{1},\text{000},\text{ Bq}/\text{kg},$$ (3)

where CPM_fon_ is the number of pulses per minute registered by the spectrometer for pure distilled water, cpm; t is the measurement time, min; the MDA of ^3^Н in plants was 0.01 kBq/kg.

The uncertainty was calculated based on the standard deviation of the recorded pulses in the sample spectrum, taking into account the measurement errors associated with laboratory instruments used throughout all stages of sample preparation. For all the activity values obtained, the uncertainty is represented as the sum of the squares of the uncertainties. The overall uncertainty accounts for measurement variability, sample preparation errors (including the uncertainty of measuring instruments and the standard sample), and calibration-related factors (the activity of the standard sample, the uncertainty of the calibration curve, etc.). The confidence interval of uncertainty was 95%.

The radionuclide determinations were subjected to quality checks. One test sample and one ‘blank’ sample were added to each batch of the analysed samples (10 samples per batch). The test sample was randomly selected from the set of samples included in the batch, whereas the ‘blank’ sample was prepared in advance using samples collected from territories with’background’ contents of technogenic radionuclides. The test and ‘blank’ samples were analysed simultaneously with the remaining samples. The test sample was intended to control the quality and repeatability of the analytical results, whereas the ‘blank’ sample was used to control hypothetical sample cross-contamination.

### 2.3 Methodology for estimating the doses of internal radiation to terrestrial biota

The dose rate of internal irradiation of terrestrial biota was calculated as follows (ICRP 108, 2008):


D=A×DCC (4)

where A is the activity concentration of a radionuclide in plants, sediments, soil, and water, used in calculating the dose rate from internal, Bq/kg; DCC is the dose factor of internal irradiation (µGy × kg/day/Bq). The values of dose coefficients are tabulated in ICRP 108 [37]. Radiation doses for terrestrial biota were calculated based on the radionuclide content in organisms.

## 3. Results and discussion

### 3.1 Concentration of ^3^Н in plants

Laboratory analyses revealed that the concentration of ^3^H in the studied plants varied within a range: TFWT from 13 to 51 kBq/kg, ОBТ from 0.7 to 15 kBq/kg (‘Degelen’ site boundary), TFWT from 0.012 to 0.18 kBq/kg, ОBТ from <0.01 to 0.091 kBq/kg (SEP), TFWT from 0.044 to 39 kBq/kg, ОBТ from 0.1037 to 13 kBq/kg (coastal area of Shagan River) (Table 1).

**Table 1:** Results of activity concentration of tritiated free water (TFWT) and organically bound ^3^H (OBT) in plants.

Site	Plants	Activity concentration of ^3^Н in plants, kBq/kg							
		May		June		July		August	
		TFWT	ОBТ	TFWT	OBT	TFWT	OBT	TFWT	OBT
Boundary of ‘Degelen’ site									
BMS-1	willow (stems)	-*	–	35 ± 4	13 ± 1	38 ± 4	12 ± 1	40 ± 4	12 ± 1
	willow (leaves)	–	–	31 ± 3	12 ± 1	42 ± 4	14 ± 1	41 ± 4	15 ± 1
	cane	–	–	31 ± 3	12 ± 1	40 ± 4	11 ± 1	40 ± 4	12 ± 1
	galatella	–	–	24 ± 3	0.88 ± 0.1	32 ± 3	7.4 ± 0.8	–	–
BMS-2	willow (stems)	–	–	28 ± 3	9.7 ± 1.0	50 ± 5	13 ± 1	17 ± 2	9.2 ± 1.0
	willow (leaves)	–	–	26 ± 3	8.6 ± 0.9	51 ± 5	14 ± 1	24 ± 2	0.8 ± 0.08
	cane	–	–	19 ± 2	7.7 ± 0.8	31 ± 3	7.3± 0.3	17 ± 2	0.7 ± 0.07
	galatella	–	–	13 ± 1	2.6 ± 0.3	–	–	–	–
BMS-3	poplar (stems)	–	–	31 ± 3	12 ± 1	34 ± 3	8.0± 0.8	29 ± 3	13 ± 1
	poplar (leaves)	–	–	19 ± 3	8.1 ± 0.8	35 ± 4	12 ± 1	30 ± 3	8.7 ± 0.9
	cane	–	–	34 ± 3	9.4 ± 1.0	35 ± 4	8.0 ± 0.8	35 ± 3	11 ± 1
	galatella	–	–	23 ± 2	5.4± 0.5	34 ± 3	8.2 ± 0.8	26 ± 3	8.7± 0.9
SEP									
BMS-4	cheegrass	0.027 ± 0.006	0.09 ± 0.036	0.024 ± 0.005	0.024 ± 0.009	0.018 ± 0.004	Below MDA	0.11 ± 0.02	0.02 ± 0.007
	mat grass	–	–	0.026 ± 0.005	Below MDA	0.023 ± 0.005	Below MDA	–	–
BMS-5	meadowsweet	0.024 ± 0.005	0.08 ± 0.028	0.025 ± 0.005	0.019 ± 0.007	0.02 ± 0.004	below MDA	0.04 ± 0.007	0.033 ± 0.009
	mat grass	–	–	0.015 ± 0.003	Below MDA	0.023 ± 0.005	Below MDA	0.064± 0.01	0.033 ± 0.009
BMS-6	cheegrass	0.012 ± 0.003	0.01 ± 0.006	0.018 ± 0.004	0.035 ± 0.011	0.025 ± 0.005	Below MDA	0.05 ± 0.009	0.035 ± 0.009
	mat grass	–	–	0.014 ± 0.003	Below MDA	0.024 ± 0.005	Below MDA	0.18± 0.02	0.051 ± 0.011
Coastal area of Shagan River									
BMS-7	cane	–	–	0.69 ± 0.08	0.30 ± 0.04	0.53 ± 0.05	0.2 ± 0.03	0.58 ± 0.06	0.33 ± 0.04
	wild rye	0.044 ± 0.007	0.21 ± 0.05	0.43 ± 0.05	0.20 ± 0.03	1.4 ± 1	0.2 ± 0.03	0.36 ± 0.04	0.41 ± 0.05
	cheegrass	–	–	0.5 ± 0.06	0.18 ± 0.03	1.2 ± 1.0	0.19 ± 0.03	1.2 ± 0.1	0.28 ± 0.04
BMS-8	cane	–	–	6.7 ± 0.7	2.5 ± 0.3	39 ± 4	10 ± 1	27 ± 3	13 ± 1
	wild rye	1.4 ± 0.1	2.3 ± 0.3	3.3 ± 0.3	2.0 ± 0.2	13 ± 1	1.9 ± 0.2	8.6 ± 0.9	7.7 ± 0.8
	cheegrass	–	–	1.6 ± 0.2	1.5 ± 0.2	13 ± 1	1.1 ± 0.1	3.7 ± 0.4	2.3 ± 0.2
BMS-9	cane	–	–	0.49 ± 0.05	0.18 ± 0.03	1.4 ± 1.0	0.39 ± 0.05	0.73 ± 0.08	0.24 ± 0.03
	wild rye	0.12 ± 0.02	0.14 ± 0.05	0.19 ± 0.02	0.13 ± 0.02	0.28 ± 0.03	0.051 ± 0.013	0.14 ± 0.02	0.044 ± 0.011
	cheegrass	–	–	0.19 ± 0.02	0.085 ± 0.016	0.14 ± 0.02	0.037 ± 0.011	0.19 ± 0.02	0.069 ± 0.014

-* – no data available

The activity concentration of ^3^H in plants varied across the widest range (up to 3 orders of magnitude) depending on the sampling plot, compared with ranges of values for different plant species and for different sampling periods. The peak ^3^H activity concentration (up to 51 kBq/kg) was recorded for plants collected at the boundary of the ‘Degelen’ site, which is characterised by the highest ^3^H content in the abiotic components of the environment [30]. As expected, the minimum values of activity concentration recorded at SEP did not exceed 0.18 kBq/kg.

Minor variations in the ^3^H content in plants were typical for different sites, plant species, and sampling times. Differences in the ^3^H content in various organs of woody plants (willows and poplar) collected at the border of the ‘Degelen’ site were, in most cases, within the uncertainty range (Table 1).

### 3.2 TFWT and OBT measurements in vegetation

Surface water containing ^3^H (HTO) readily exchanges hydrogen with atmospheric moisture, precipitation, and soil moisture. Some of the ^3^H enters plants from the atmosphere through leaves and is also absorbed by the roots from the soil. ^3^H in the TFWT form is distributed throughout the fresh plant tissue and subsequently incorporated into organic compounds, forming OBT. The presence of surface water, such as creeks along the boundary of the ‘Degelen’ site, the Shagan River riverbed, and the groundwater flows that feed them, ensures the continuity of this process. Further, the behaviour of ^3^H in terrestrial plants mainly depends on their use of water [38]. As can be seen from Table 1, the concentration of TFWT in the studied plants at the border of the ‘Degelen’ site and in the coastal area of the Shagan River is consistently higher than OBT. The studied reservoirs in the conditionally ‘background’ area may dry out seasonally, while the differences between the concentrations of TFWT and OBT remain relatively minor.

The OBT concentration represents a long-term accumulation of ^3^H in plants from several weeks to several decades [39]. Therefore, this parameter is relatively stable. In most instances, no significant variation in ^3^H content was observed between different species or across the research period (Table 1). The TFWT concentration reflects the short-term uptake of ^3^H by plants [40] through water (both surface and groundwater) containing elevated ^3^H levels [29]. Changes in TFWT content in various plant species over the entire research period are presented as ratios relative to the maximum activity concentration for each research site at the boundary of the ‘Degelen’ site (Fig 3a, 3c, 3e) and along the coastal area of the Shagan River (Fig 3b, 3d, 3f).

As can be seen from Fig 3, the maximum average TFWT values both at the border of the ‘Degelen’ site and along the coastal area of the Shagan River were observed in July. This difference in TFWT concentrations is most significant for the BMS-2 site, although the HTO in surface water decreases for this plot during the entire research period (unlike BMS-1 and BMS-3) [29]. This may be due to higher concentrations of ^3^H in the groundwater in the research area [23,41]. A higher concentration of OBT and TFWT (Table 1) at the boundary of the ‘Degelen’ site was observed for phreatophyte plants (cane, poplar, and willow). In contrast, comparatively lower concentrations were observed for galatella, which also indicates a higher concentration of ^3^H in groundwater than in surface water [20].

The correlation between ^3^H concentration in plants and surface water is clearly observed in the riparian zone of the Shagan River. The highest OBT values were observed in cane (Table 1), which grows in water, indicating high values of HTO in water. This was confirmed by TFWT concentrations in cane from the BMS-8 and BMS-9 sites, where an increase in HTO levels was observed across the research period [29]. Simultaneously, the HTO content in surface water at the BMS-7 site remained relatively constant on average. Meanwhile, higher ^3^H concentrations in the soil at the BMS-7 site were observed in the lower horizons [29], which may indicate higher ^3^H concentrations in groundwater. Higher TFWT values at the BMS-7 site were observed in wild rye and cheegrass growing on land.

^3^H concentrations in plants in the SEP (Table 1) were unstable. In many cases, OBT concentrations were below the detection limit of the analytical instruments and methodological framework used.

Significant increase in the TFWT concentration in the SEP was noted only in August. During the same period, measurable OBT values were recorded in all collected plant samples. Meanwhile, surface water in reservoirs in this area was present only in July; however, the HTO concentrations in these samples were below the detection limit of the analytical instruments and methodological framework used [29]. In this case, groundwater may be the primary source of ^3^H uptake into plants. This was confirmed by the more frequently recorded quantitative OBT values in meadowsweet and cheegrass (9 of 12 samples). Compared with mat grass, these plants obtain most of their nutrients from groundwater. The presence of ^3^H was also observed in the soil. Moreover, for the BMS-4 and BMS-5 sites, the evaluated concentrations of ^3^H were not recorded from the surface, but at a depth of 200–300 cm (Table 2), which may also indicate capillary rise from groundwater.

**Table 2:** Activity concentrations of ^3^Н species in the soil in the southeastern part of the Semipalatinsk Test Site (Timonova et al., 2024).

Site	Soil sampling depth, cm	^3^Н activity concentration, Bq/kg			
		^3^Н in surface-adsorbed water (TSA)	^3^Н in interlayer water (TIW)	Hydroxyl + organically bound ^3^Н (HT + OBT)	Crystal-line bound ^3^Н (CBT)
		AC ± SD	AC ± SD	AC ± SD	AC ± SD
BMS-4	0–100	4 ± 1	below MDA	below MDA	below MDA
	100–200	6 ± 1	below MDA	below MDA	below MDA
	200–300	30 ± 12	below MDA	220 ± 20	below MDA
BMS-5	0–100	12 ± 1	below MDA	below MDA	below MDA
	100–200	11 ± 2	below MDA	1 710 ± 170	below MDA
	200–350	8 ± 1	below MDA	1 470 ± 150	below MDA
BMS-6	0–150	22 ± 2	below MDA	1 000 ± 100	1 250 ± 130

MDA for soil of ^3^Н in surface-adsorbed water and ^3^Н in interlayer water is 0.1 Bq/kg; MDA for soil of organically bound ^3^Н and crystal bound ^3^Н is 50 Bq/kg.

Currently, several studies have reported the OBT/TFWT ratios in plants [40,42-43]. However, existing studies are mostly based on background levels, often near NPPs, and under conditions without an open tritium ^3^H source. Table 3 presents the OBT/TFWT ratios for the research plots at the STS.

**Table 3:** OBT/TFWT ratio for plants from all research plots.

Research plot	n	Range	Arithmetic mean ± SD	Geometric mean ± Geometric SD
Boundary of the ‘Degelen’ site	33	0.033–0.54	0.30 ± 0.019	0.26 ± 0.11
SEP	8	0.21–1.9	0.77 ± 0.19	0.62 ± 0.55
Coastal area of the Shagan River	27	0.085–1.1	0.43 ± 0.049	0.36 ± 0.26

SEP, southeastern part of the Semipalatinsk Test Site; SD, standard deviation.

The average OBT/TFWT value at the boundary of the ‘Degelen’ site was 0.3, which is comparable to data previously obtained in this area [21]. Slightly higher OBT/TFWT values were recorded in the coastal zone along the Shagan River (0.4). Although OBT persists in the environment longer than HTO [32], the presence of surface and groundwater flows with high ^3^H content in the research area results in higher TFWT activity concentrations. Comparatively higher OBT/TFWT values were observed in the SEP (0.7). Meanwhile, all obtained OBT/TFWT values are generally significantly lower than those reported in literature. So, the mean OBT/ TFWT ratios for vegetation samples collected near Canadian nuclear facilities ranged from 1.0 ± 1.7 to 2.2 ± 2.6 [43], the OBT/TFWT values for in terrestrial plants at Tarapur (a tropical NPP Site in India) were found to be 4.63 for shrubs and 3.66 for trees [44].

### 3.3. Assessment of a potential vegetation exposure to ^3^H

Based on the results of ^3^H concentrations in plants, an assessment of the internal dose rate was conducted for all studied species at each research site. A comparative analysis of the internal dose rate for different plant species across the research plots of the STS is presented in Fig 4.

The histograms (Fig 4) show that the internal dose rates from ^3^H vary between plant species and are generally determined by their relationship with water. In particular, the comparatively highest ^3^H concentrations and internal dose rates were typically observed in hygrophytes (cane) and phreatophytes (willow, poplar, wild rye, and cheegrass), depending on the ^3^H content in surface and groundwater, respectively. Lower internal dose rates were recorded for meadow (galatella) and steppe (mat grass) ecosystem species, whose water intake is primarily derived from precipitation. Within the research area, the maximum internal dose rates were recorded for woody species (willow and poplar) at the ‘Degelen’ site boundary at up to 1.2 × 10^−2^ µGy/day. The minimum doses, as expected, were observed in xerophytic species (mat grass) in the SEP, with doses ranging from 6.3 × 10 ⁻ ⁶ to 2.4 × 10 ⁻ ⁵ µGy/day.

Comparatively higher dose rates in the coastal zone of the Shagan River were observed at the BMS-8 site, which is characterised by elevated ^3^H concentrations in all environmental compartments [29]. At the same time, the internal dose rates measured in the studied plant species remained well below the threshold levels for radiological impact on biota. According to literature [37,45], the radiation effects scale covers a wide range of irradiation levels from 10−6–1 Gy/day and a wide range of radiation effects – from stimulation to acute radiation injury. The obtained results for the internal irradiation dose rate of the studied plants are significantly below the safe level of radiation exposure for biota, indicating the absence of a threat (danger) to the ecosystem of the study area.

### Conclusion

Here, the concentrations of TFWT and OBT were established for individual plots in the STS with different types of radioactive contamination, complementing previous studies [29]. In most cases, TFWT concentrations in the studied plants were higher than those of OBT: TFWT from 13 to 51 kBq/kg, OBT from 0.7 to 15 kBq/kg (boundary of the ‘Degelen’ site), TFWT from 0.012 to 0.18 kBq/kg, OBT from <0.019 to 0.088 kBq/kg (SEP), TFWT from 0.044 to 39 kBq/kg, OBT from 0.037 to 13 Bq/kg (coastal area along the Shagan River). The OBT/TFWT ratios (from 0.3 to 0.7) deviated from those obtained by other researchers [43,44]. The internal radiation dose rates of the studied plants (from 6.3 × 10^−6^ to 1.2 × 10^−2^ µGy/day) were significantly below the established safety threshold for radiation exposure to biota.

## Supporting information

S1 Appendix A copy of file (in Word format) is provided in the appendix as figures. Figures includes details of research areas, stages of sample preparation and measuring equipment used for this review.
(DOC)

## References

[1] Buesseler KO. Opening the floodgates at Fukushima. Science. 2020;369(6504):621–2. doi: 10.1126/science.abc1507 32764053

[2] IAEA. Transfer of tritium in the environment after accidental releases from nuclear facilities: report of working group 7 Tritium accidents of EMRAS II topical heading approaches for assessing emergency situations. Vienna: International Atomic Energy Agency; 2014.

[3] Momoshima N. Tritium in the environment. Radiat Prot Dosimetry. 2022;198(13–15):896–903. doi: 10.1093/rpd/ncac002 36083730

[4] UNSCEAR. Sources and effects of ionizing radiation, exposures to the public from man-made sources of radiation (Annexe C, Comite scientifique des Nations Unies pour l’ Etude des Effets des Rayonnements Ionisants UNSCEAR). 2000. 134

[5] UNSCEAR. Sources, effects and risks of ionizing radiation. In: Biological effects of selected internal emitters—Tritium. United Nations Scientific Committee on the Effects of Atomic Radiation; 2016. 122.

[6] Park T-K, Kim S-K. Tritium: its generation and pathways to the environment at CANDU 6 generating stations. Nuclear Eng Design. 1996;163(3):405–11. doi: 10.1016/0029-5493(96)01157-0

[7] Song MJ, Son SH, Jang CH. Tritium inventory prediction in a CANDU plant. Waste Manag. 1995;15(8):593–8. doi: 10.1016/0956-053x(96)00017-7

[8] Dolin V, Yakovlev Y, Cancemi SA, Lo Frano R. The impact of tritium in the environment. Appl Sci. 2025;15(12):6664. doi: 10.3390/app15126664

[9] Askerbekov S, Kenzhina I, Kulsartov T, Chikhray Ye, Tazhibayeva I, Ponkratov Yu, et al. Analysis of reactor experiments to study the transfer processes of generated tritium in lithium cps (capillary-porous system). Inter J Hydrog Energy. 2022;47(11):7368–78. doi: 10.1016/j.ijhydene.2021.03.163

[10] Zholdybayev T, Shaimerdenov A, Kulsartov T, Kharkin P, Milts O, Askerbekov S, et al. Method of measurement of residual tritium in irradiated lithium ceramics. J Phys: Conf Ser. 2025;3089(1):012014. doi: 10.1088/1742-6596/3089/1/012014

[11] Kulsartov T, Kenzhina I, Chikhray Ye, Zaurbekova Zh, Kenzhin Ye, Aitkulov M, et al. Determination of the activation energy of tritium diffusion in ceramic breeders by reactor power variation. Fusion Eng Design. 2021;172:112783. doi: 10.1016/j.fusengdes.2021.112783

[12] Kulsartov T, Kenzhin Y, Knitter R, Kizane G, Chikhray Y, Shaimerdenov A, et al. Investigation of hydrogen and deuterium impact on the release of tritium from two-phase lithium ceramics under reactor irradiation. Nucl Mat Energy. 2022;30:101115. doi: 10.1016/j.nme.2022.101115

[13] Kulsartov T, Kenzhina I, Knitter R, Leys J, Zaurbekova Z, Shaimerdenov A, et al. Influence of various gases and water vapors on the processes of tritium release from two-phase lithium ceramics. Fusion Eng Design. 2024;202:114302. doi: 10.1016/j.fusengdes.2024.114302

[14] Ichimasa M, Ichimasa Y, Azuma Y, Komuro M, Fujita K, Akita Y. Oxidation of molecular tritium by surface soils. J Radiat Res. 1988;29:144–51. 3172040 10.1269/jrr.29.144

[15] Panitskiy A, Bazarbaeva A, Baigazy S, Alexandrovich I, Larionova N. Radioecological characteristics of Siberian roe deer (*Capreolus pygargus Pal.*, 1771) inhabiting locations of nuclear weapon tests. PLoS One. 2024;19(9):e0308518. doi: 10.1371/journal.pone.0308518 39288116 PMC11407653

[16] Krivitskiy PY, Larionova NV, Monayenko VN, Subbotin SB, Chernov AA, Panitskiy AV. Peculiarities of radioactive soil contamination in places of underground nuclear tests in the Semipalatinsk test site. J Environ Radioact. 2022;253–254:106991. doi: 10.1016/j.jenvrad.2022.106991 36084569

[17] Larionova N, Toporova A, Krivitskiy P, Polevik V, Lechshenko N, Monayenko V, et al. Artificial radionuclides in the plant cover around nuclear fuel cycle facilities. PLoS One. 2024;19(7):e0306531. doi: 10.1371/journal.pone.0306531 38954696 PMC11218991

[18] Kunduzbayeva AY, Lukashenko SN, Kabdyrakova AM, Larionova NV, Magasheva RY, Bakirova GA. Speciation of 137Cs, 90Sr, 241Am, and 239 + 240Pu artificial radionuclides in soils at the Semipalatinsk test site. J Environ Radioact. 2022;249:106867. doi: 10.1016/j.jenvrad.2022.106867 35523044

[19] Lyakhova ON, Lukashenko SN, Larionova NV, Tur YS. Contamination mechanisms of air basin with tritium in venues of underground nuclear explosions at the former Semipalatinsk test site. J Environ Radioact. 2012;113:98–107. doi: 10.1016/j.jenvrad.2012.02.010 22672895

[20] Larionova NV, Lukashenko SN, Lyakhova ON, Aidarkhanov AO, Subbotin SB, Yankauskas AB. Plants as indicators of tritium concentration in ground water at the Semipalatinsk test site. J Environ Radioact. 2017;177:218–24. doi: 10.1016/j.jenvrad.2017.06.032 28711773

[21] Timonova LV, Lyakhova ОN, Aidarkhanov АО, Serzhanova ZB. Tritium contaminated soil in sites for Semipalatinsk above-ground nuclear tests. Radiat Risk. 2020;29(4):106–17. doi: 10.21870/0131-3878-2020-29-4-106-117

[22] Larionova NV, Krivitskiy PY, Aidarkhanova AK, Polevik VV, Timonova LV, Monayenko VN, et al. Tritium content in vegetation cover at nuclear test locations at the “Sary-Uzen” site in the Semipalatinsk Test Site. Ecotoxicol Environ Saf. 2024;288:117387. doi: 10.1016/j.ecoenv.2024.117387 39581115

[23] Larionova N, Timonova L, Toporova A, Aidarkhanova A. Tritium distribution in environmental compartments of the impact zone of radioactively contaminated areas. Ecol Indicat. 2025;175:113567. doi: 10.1016/j.ecolind.2025.113567

[24] Yankauskas AB, Larionova NV, Shatrov AN. Effect of tritium on the morpho-anatomical structure of the common reed (Phragmites australis). Radiat Risk. 2021;30(2):133–45. doi: 10.21870/0131-3878-2021-30-2-133-145

[25] Aidarkhanova A, Larionova N, Tashekova A, Dyussembayeva M, Mamyrbayeva A, Timonova L, et al. Assessment of the radionuclide and chemical composition of the Irtysh River water at the Republic of Kazakhstan territory. RSC Adv. 2024;14(36):26208–18. doi: 10.1039/d4ra02557a 39165791 PMC11334156

[26] Genova SV, Lukashenko SN, Aidarkhanov AO. Research of the nature and levels of radionuclide contamination of the Shagan River waters (results of 2010). In: Topical issues of radioecology of Kazakhstan, proceedings of the IRSE NNC RK for 2010, 2011. 165–78.

[27] Knatova MK, Umarov MA, Burkitbaev MM, Vintro LL, Mitchell PI, Priest N. Radionuclide analysis of water samples from the former Semipalatinsk test site. In: News of the National sciences academy of the republic of Kazakhstan. 2006;6:41–6.

[28] Sudprasert W, Khamanek K, Khuntong S, Tanyasit J, Saenboonruang K, Toyen D, et al. Baseline tritium measurements in Thailand’s water bodies: supporting sustainable nuclear energy development. J Environ Radioact. 2025;282:107604. doi: 10.1016/j.jenvrad.2024.107604 39708542

[29] Timonova L, Larionova N, Aidarkhanova A, Lyakhova O, Aktayev M, Serzhanova Z, et al. Tritium distribution in the “water-soil-air” system in the Semipalatinsk Test Site. PLoS One. 2024;19(4):e0297017. doi: 10.1371/journal.pone.0297017 38573885 PMC10994305

[30] Melintescu A, Patryl L. Interception and uptake by plants leaves of tritium from precipitation. J Environ Radioact. 2025;285:107677. doi: 10.1016/j.jenvrad.2025.107677 40120230

[31] Meng D, Wang W, Du Y, Xiao C, Wen W, Dan G, et al. Tritium distribution in typical plants around tritium laboratory in south-west of China. J Environ Radioact. 2021;227:106504. doi: 10.1016/j.jenvrad.2020.106504 33307328

[32] Kim SB, Bredlaw M, Rousselle H, Bond M, Stuart M. Determination of the baseline tritium concentrations (HTO, TFWT and OBT) in soil and plants in Ontario, Canada. J Environ Radioact. 2022;243:106810. doi: 10.1016/j.jenvrad.2021.106810 34990898

[33] Aktayev M, Subbotin S, Aidarkhanov A, Aidarkhanova A, Timonova L, Larionova N. Characterization of geological and lithological features in the area proximal to tritium-contaminated groundwater at the Semipalatinsk test site. PLoS One. 2024;19(3):e0300971. doi: 10.1371/journal.pone.0300971 38517930 PMC10959336

[34] Batyrbekov E, Aidarkhanov A, Vityuk V, Larionova N, Umarov M. Comprehensive radioecological survey of Semipalatinsk Test Site, Kurchatov, Kazakhstan. 2021;339.

[35] Device for the extraction of water from the samples. Astana: KazInSt Publishing; 2015.

[36] ISO. Water quality. Tritium. A liquid scintillation counting technique to determine activities. 2019.

[37] ICRP. Environmental protection: the concept and use of reference animals and plants. 2008.

[38] Tritium in the environment. Technical reports series. International Atomic Energy Agency; 2025. doi: 10.61092/iaea.0qjt-xo5o

[39] Kim SB, Baglan N, Davis PA. Current understanding of organically bound tritium (OBT) in the environment. J Environ Radioact. 2013;126:83–91. doi: 10.1016/j.jenvrad.2013.07.011 23962797

[40] Melintescu A, Galeriu D. Uncertainty of current understanding regarding OBT formation in plants. J Environ Radioact. 2017;167:134–49. doi: 10.1016/j.jenvrad.2016.11.026 27916298

[41] Mikhailov AV, Lukashenko SN, Thomson AV, Edomskaya MA. Investigation of tritium content in wild plants growing in an area with an underground source of tritium. J Environ Radioact. 2025;282:107602. doi: 10.1016/j.jenvrad.2024.107602 39708541

[42] Korolevych VY, Kim SB, Davis PA. OBT/HTO ratio in agricultural produce subject to routine atmospheric releases of tritium. J Environ Radioact. 2014;129:157–68. doi: 10.1016/j.jenvrad.2013.12.014 24502954

[43] Thompson PA, Kwamena N-OA, Ilin M, Wilk M, Clark ID. Levels of tritium in soils and vegetation near Canadian nuclear facilities releasing tritium to the atmosphere: implications for environmental models. J Environ Radioact. 2015;140:105–13. doi: 10.1016/j.jenvrad.2014.11.009 25461522

[44] Baburajan A, Gaikwad RH, Varakhedkar VK, Saradhi IV, Vinod Kumar A. Assessment of TFWT and OBT in terrestrial plants at Tarapur, a tropical NPP Site in India, and a comparative analysis with model-computed concentrations. J Radioanal Nucl Chem. 2025;334(8):5771–83. doi: 10.1007/s10967-025-10313-w

[45] Kryshev II, Sazykina TG. Radiation safety of the environment: necessity of harmonize Russian and international regulatory and methodological documents, taking into account the requirements of federal legislation and the new international basic safety standards ONB-2011. Radiat Risk. 2013;22(1):47–61.

