# Supplementary material for: Tritium in vegetation at various types of radioactive contamination sites under arid climate conditions
Source: PLoS One. 2026 Jan 27;21(1):e0339645. doi: 10.1371/journal.pone.0339645 (PMC12843519; doi:10.1371/journal.pone.0339645)
Supplement: S1 Appendix — Figures includes details of research areas, stages of sample preparation and measuring equipment used for this review. (DOC) [file pone.0339645.s001.doc]

**S1 Appendix**

| 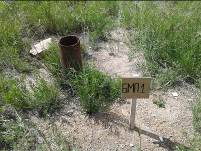 | 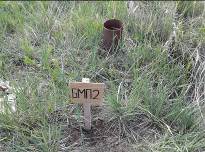 | 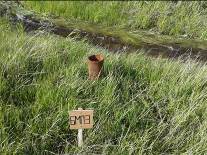 |
| --- | --- | --- |

**Fig 1. Research areas at the ‘Degelen’ site.**

| 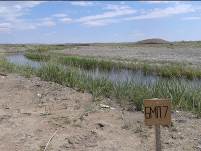 | 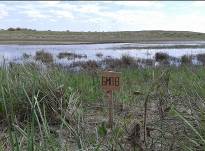 | 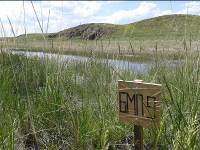 |
| --- | --- | --- |

**Fig 2. Research areas in the riverside zone of the Shagan.**

| 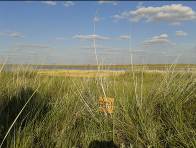 | 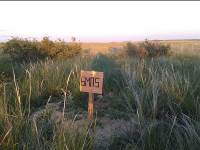 | 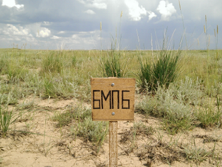 |
| --- | --- | --- |

**Fig 3. Research areas on the conventionally ‘background’ area.**

| 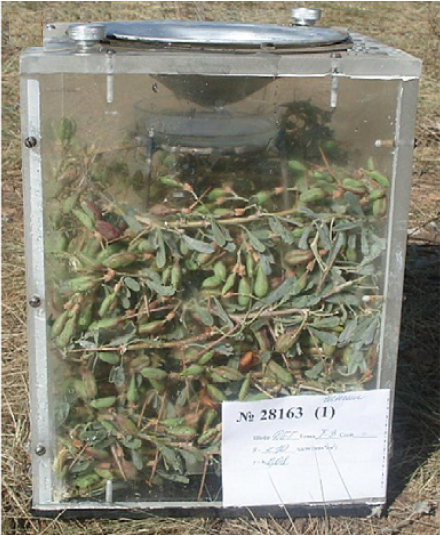 | 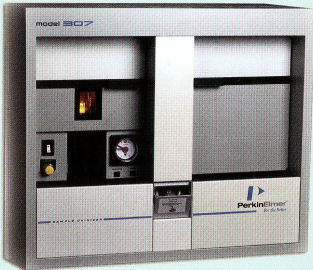 |
| --- | --- |
| a. Extraction of water from plant samples | b. ‘Sample Oxidizer A307’ sample preparation system |

**Fig 4. Sample preparation.**

| 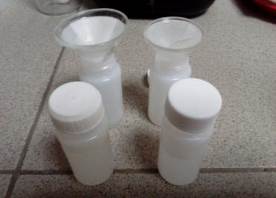 | 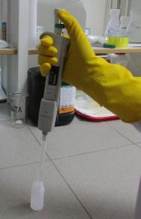 |
| --- | --- |
| a) Filtration of liquid samples | b) Aliquot selection |
| 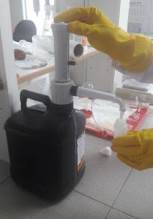 | 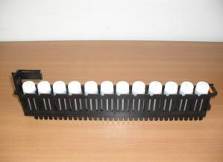 |
| c) Adding a scintillator | d) Prepared samples for measurement |

**Fig 5.** **Stages of sample preparation for measuring tritium concentration.**


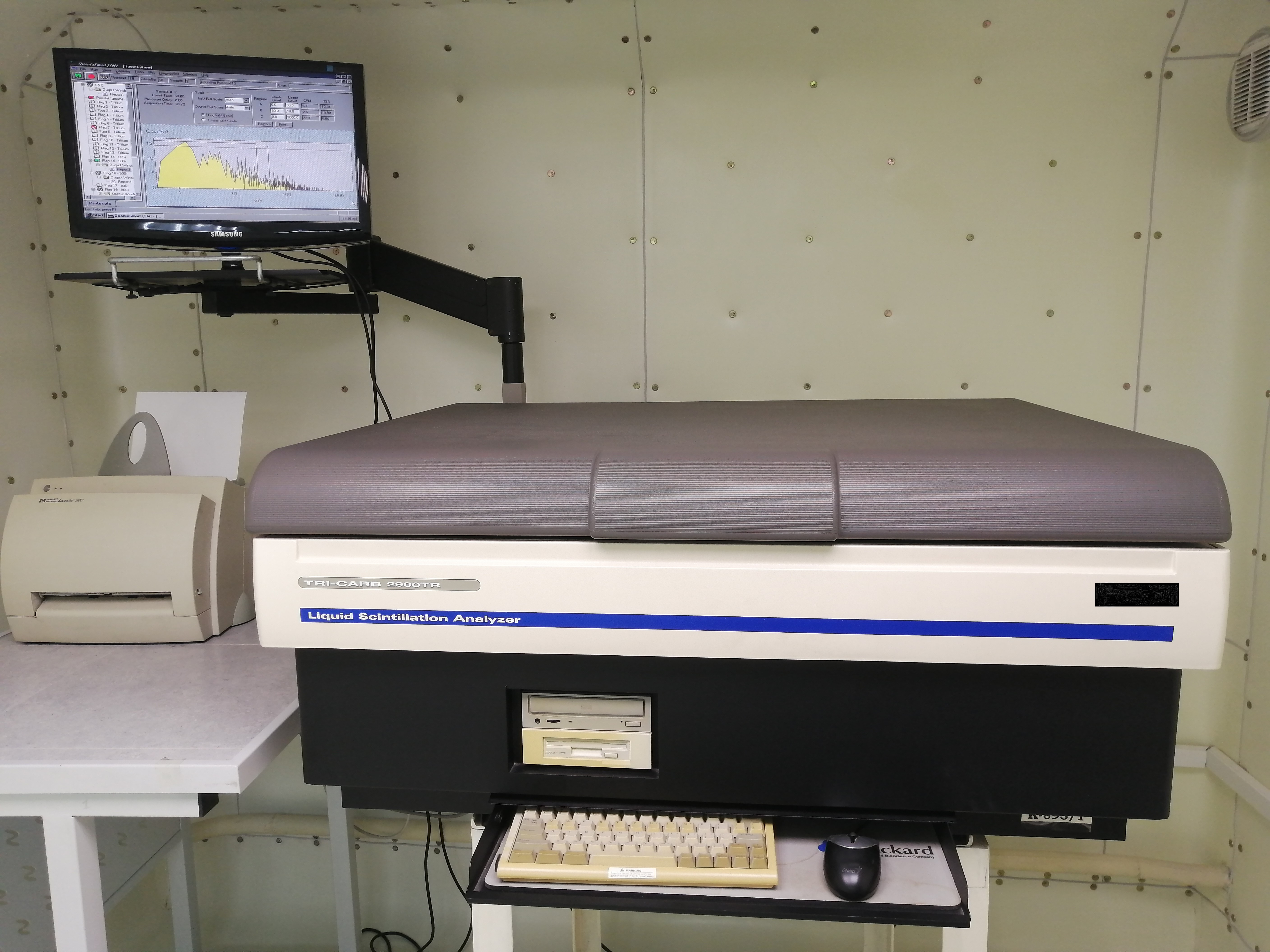


**Fig 6. Liquid scintillation spectrometer ‘TRI-CARB 2900 TR’.**
